# Identification and characterization of *PsFwC9* conferring Fusarium wilt resistance in pea

**DOI:** 10.1093/hr/uhag166

**Published:** 2026-04-23

**Authors:** Dong Deng, Wenqi Wu, Canxing Duan, Ming Feng, Renfeng Xue, Weide Ge, Suli Sun, Zhendong Zhu

**Affiliations:** Key Laboratory of Grain Crop Genetic Resources Evaluation and Utilization, Institute of Crop Sciences, Chinese Academy of Agricultural Sciences, Beijing 100081, China; Crop Research Institute, Liaoning Academy of Agricultural Sciences, Shenyang 110161, China; Key Laboratory of Grain Crop Genetic Resources Evaluation and Utilization, Institute of Crop Sciences, Chinese Academy of Agricultural Sciences, Beijing 100081, China; Key Laboratory of Grain Crop Genetic Resources Evaluation and Utilization, Institute of Crop Sciences, Chinese Academy of Agricultural Sciences, Beijing 100081, China; Crop Research Institute, Liaoning Academy of Agricultural Sciences, Shenyang 110161, China; Crop Research Institute, Liaoning Academy of Agricultural Sciences, Shenyang 110161, China; Crop Research Institute, Liaoning Academy of Agricultural Sciences, Shenyang 110161, China; Key Laboratory of Grain Crop Genetic Resources Evaluation and Utilization, Institute of Crop Sciences, Chinese Academy of Agricultural Sciences, Beijing 100081, China; Key Laboratory of Grain Crop Genetic Resources Evaluation and Utilization, Institute of Crop Sciences, Chinese Academy of Agricultural Sciences, Beijing 100081, China

## Abstract

Pea (*Pisum sativum* L.) is one of the most important edible legumes in China, with both planting area and total yield ranking among the highest in the world. Fusarium wilt, caused by *Fusarium oxysporum* f. sp. *pisi* (*Fop*), is a severe factor limiting pea production. The deployment of resistant pea cultivars is the most effective and sustainable strategy for controlling this disease. In the present study, a novel resistance gene *PsFwC9*, conferring resistance to *Fop* race 5, was identified in the resistant pure line Chengwan 9-8 (CW9-8), and its candidate gene *Psat4g213640* was characterized and functionally validated to be associated with disease resistance. Genetic analysis of the F₂ population derived from the cross between the resistant parent CW9-8 and the susceptible parent Chengwan 9-1 (CW9-1) revealed that *PsFwC9* was controlled by a single dominant gene. Based on whole-genome resequencing, bulked segregant analysis sequencing (BSA-seq) and fine mapping, *PsFwC9* was localized to an 817.06-kb region on chromosome 4 (i.e. linkage group IV, chr4LG4), flanked by KASP markers A016508 and A016511, and co-segregated with four markers. Haplotype analysis revealed that only the marker A016615 was significantly associated with Fusarium wilt resistance, and this marker was designated as a diagnostic marker for *PsFwC9*. Marker A016615 was located at 425 699 725 bp on chr4LG4, corresponding to the 277 bp within *Psat4g213640*, where a ‘A/G’ single-nucleotide polymorphism caused an amino acid substitution leading to an alteration in protein structure; therefore, *Psat4g213640* was identified as the *PsFwC9* candidate gene. Quantitative real-time PCR analysis showed no significant difference in the expression levels of *Psat4g213640* between CW9-8 and CW9-1. Overexpression of the candidate gene *Psat4g213640^CW9-8^* in the hairy root system significantly enhanced the resistance of CW9-1 to Fusarium wilt, whereas RNA interference-mediated silencing of *Psat4g213640^CW9-8^* reduced the resistance of CW9-8, indicating that *Psat4g213640^CW9-8^* played a crucial role in pea resistance to Fusarium wilt. In addition, subcellular localization showed that the protein encoded by *Psat4g213640* was targeted to the endoplasmic reticulum. Collectively, these findings not only enriched the gene resources for disease resistance in pea and provided an important foundation for elucidating the molecular mechanism of *PsFwC9*-mediated resistance, but also provided important technical support for the practical application of molecular breeding for disease resistance in pea.

## Introduction

Pea (*Pisum sativum* L.), comprising green and dry pea, serves as an important edible legume for human consumption or animal feed, offering essential proteins, carbohydrates, vitamins, dietary fiber, minerals and other bioactive compounds [1–4]. Another important property of pea is its symbiotic nitrogen-fixing ability, which is one of the leading leguminous plants in agricultural systems. Since the 20th century, pea has been widely grown in the world for economic return, for soil fertility improvement in cereal and oilseed crop rotations, as a break crop for disease control and as an alternative to summer fallow [5–7].

However, the disease has become increasingly prominent with the expansion of pea cultivation area. Fusarium wilt, caused by *Fusarium oxysporum* f. sp. *pisi* (*Fop*), is a devastating disease that can severely reduce yield and quality in most pea-producing regions worldwide [8–11]. The *Fop* has been physiologically differentiated into four races (1, 2, 5 and 6), and the deployment of race-specific resistant cultivars remains the most economical and effective strategy for managing Fusarium wilt [10–13]. Several studies on Fusarium wilt resistance in pea have been undertaken over the years, giving rise to the development of resistant cultivars tailored to different physiological races for crop improvement. For example, Neumann and Xue [14] identified 49 accessions with resistance to at least one physiological race, Bani *et al.* [15] found 20 accessions resistant to race 2, and Deng *et al.* [10] discovered 192 accessions resistant to race 5. These resistant accessions provide valuable genetic resources that can be utilized in the development of resistant cultivars and integrated strategies for effective Fusarium wilt management in pea.

Exploring the genetic foundation of Fusarium wilt resistance is essential for the advancement of pea breeding [16–18]. Genetic resistance to *Fop* races 1 [17, 19], 5 [16, 20, 21] and 6 [21] is conferred by single dominant genes, and to race 2 is controlled by multiple genes [18]. Each of these genes was characterized for resistance to a specific race, but several of them may also provide cross-resistance to other races [16, 18]. Despite the availability of effective linkage maps, only a limited number of Fusarium wilt resistance-related genes and quantitative trait loci (QTLs) have been identified in pea, including the complete resistance genes *Fw* [17, 22] and *Fwf* [20, 22], as well as the putative partial resistance gene *Fnw4.1* [18], which are mapped to linkage groups (LG) 3, 6 and 4, respectively. In recent years, bulked segregant analysis sequencing (BSA-seq) has rapidly emerged as a powerful tool for detecting candidate regions associated with resistance traits and has been successfully applied to identify Fusarium wilt resistance genes in crop species, such as flax, eggplant and pea [16, 23, 24]. In pea, Deng *et al.* [16] employed BSA-seq analysis and fine mapping to identify *Psat6g003960* on chromosome 6 (linkage group II, chr6LG2) as the candidate gene for the Fusarium wilt resistance gene *FwS1* in the cultivar Shijiadacaiwan 1, and further confirmed that 88 out of 152 resistant individuals carried the *FwS1*. Nevertheless, *FwS1* may be closely linked to *Fwf* or even represent the same gene, and additional verification remains necessary [16, 20]. Besides, the complex NB-ARC (nucleotide-binding adaptor shared by APAF-1, R proteins and CED-4) repeat structure of the *FwS1* candidate gene poses challenges to investigating its functional characterization [16, 25, 26]. Therefore, discovering new resistance genes is essential for resistance breeding and elucidating the genetic foundation of Fusarium wilt resistance in pea.

In China, pea is primarily grown in mountainous or hilly areas with poor soils or as a rotational crop following major staple crops [4, 5, 27, 28]. In spite of these constraints, China holds the top position globally in green pea production and ranks third in dry pea production [29]. However, pea yields in main production regions affected by Fusarium wilt are typically reduced by 30%, with severe cases leading to losses exceeding 50% or even total crop failure [5, 10, 28]. Research on resistance genes and the genetic basis of Fusarium wilt resistance in China remains relatively limited. Chengwan 9 is an excellent green pea cultivar bred by Sichuan Academy of Agricultural Sciences (SAAS) in 2007. Our previous research revealed that the cultivar was segregant for Fusarium wilt resistance to race 5 and did not carry the *FwS1* [16]. Hence, in this study, we employ the Fusarium wilt-resistant and susceptible pure lines, Chengwan 9-8 (CW9-8, resistant) and Chengwan 9-1 (CW9-1, susceptible), derived from cultivar Chengwan 9 (CW9) to construct a genetic population to explore the Fusarium wilt resistance gene *PsFwC9* in Chengwan 9. Our objectives are to (a) identify single-nucleotide polymorphisms (SNPs) and insertion–deletions (InDels) associated with Fusarium wilt resistance, (b) identify potential resistance genes within the candidate region and (c) conduct preliminary functional validation of these candidate genes.

## Results

### Phenotypic evaluation for Fusarium wilt

**Figure 1:**
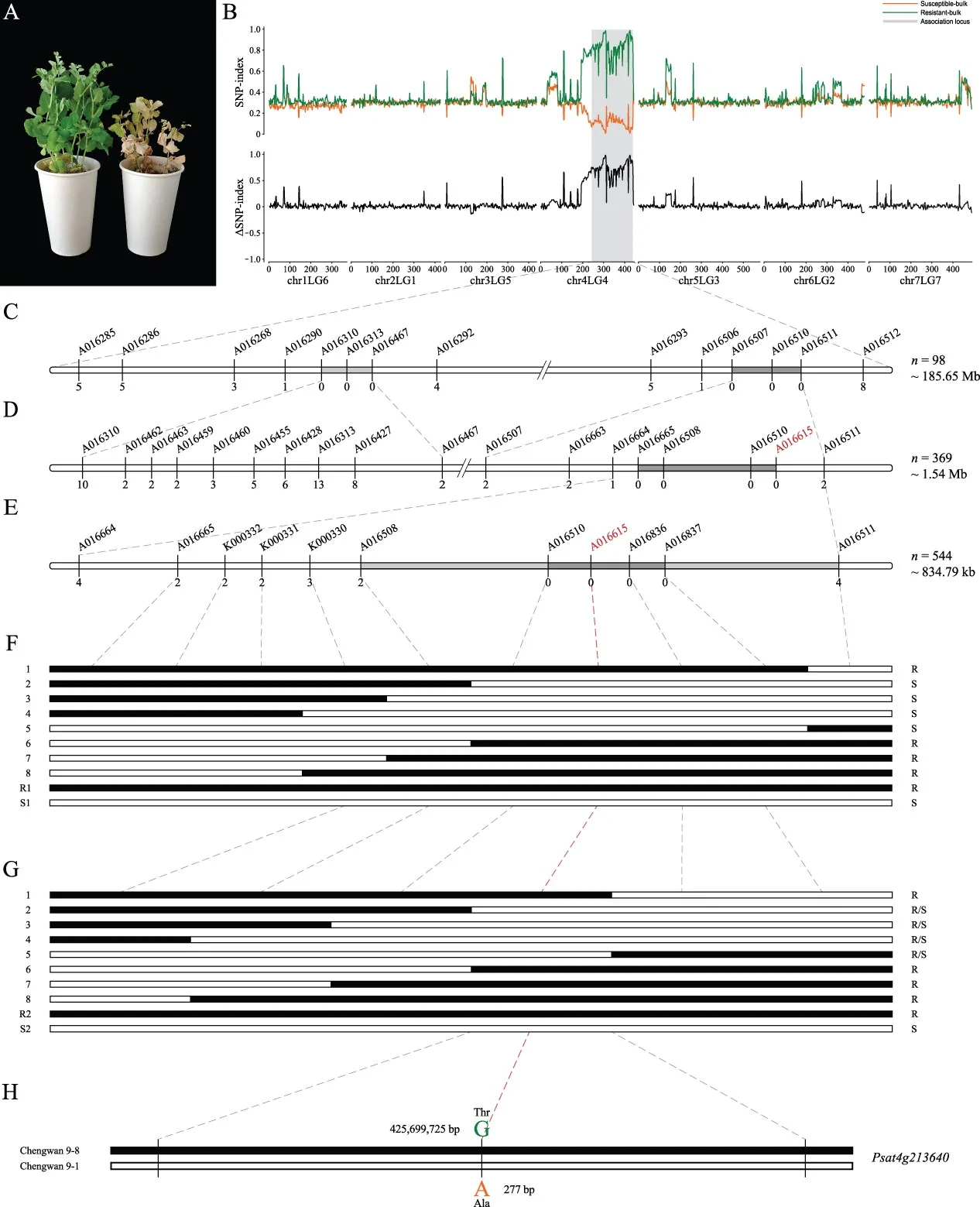
Bulked segregant analysis sequencing, mapping and haplotype analyses of the resistance gene *PsFwC9* against Fusarium wilt. (A) Phenotypes of the resistant parent Chengwan 9-8 (CW9-8, left) and the susceptible parent Chengwan 9-1 (CW9-1, right). (B) Mapping of the *PsFwC9* resistance locus through BSA-seq, with the x-axis indicating the physical positions of 7 pea chromosomes (in megabases, Mb), and the y-axis representing SNP index or ΔSNP index values. (C) The initial mapping of *PsFwC9* using 98 F_2_ individuals from the cross of CW9-1 and CW9-8. (D) The mapping of *PsFwC9* using 369 F_2_ individuals derived from CW9-1 and CW9-8. (E) Fine mapping of *PsFwC9* using 544 F_2_ individuals from the cross of CW9-1 and CW9-8. In (C), (D) and (E), the numbers below the line indicated recombinants detected by the marker. (F) Genotypes and phenotypes of key recombinants in 544 F_2_ individuals. (G) Haplotype analysis of 218 accessions showing genotypes and phenotypes of significant recombinants. In (F) and (G), black rectangles correspond to homozygous or heterozygous genotypes of CW9-8, and white rectangles represent homozygous genotypes of CW9-1. (H) Comparison of the candidate SNP locus at KASP marker A016615 between CW9-8 and CW9-1, with the vertical line marking the nonsynonymous SNP and amino acid change. The numbers above and below the rectangles represent the position on the chromosome and gene, respectively.

After 28 dpi, all seedlings of susceptible parent CW9-1 and the differential cultivars Litter Marvel, Darkskin Perfection, New Era and New Season wilted and died, whereas the plants of resistant parent CW9-8 and the differential cultivars WSU 23, WSU 28 and WSU 31 maintained normal growth with negligible observable disease symptoms (Fig. 1A). The results confirmed that CW9-8 was resistant to *Fop* race 5.

In the mapping population consisting of 544 individuals from nine CW9-1 × CW9-8 F_2_ populations, 396 individuals exhibited resistance (R) to *Fop* race 5 isolates PF22b, while 148 individuals showed susceptibility (S), corresponding to a segregation ratio of 3:1 by the Chi-squared test with *χ*^2^ = 1.78 and *P* = .18 (Table 1). The analysis of the nine F_2_ populations also revealed a consistent 3:1 segregation ratio for resistance and susceptibility, where *χ*^2^ values were below 3.84 and *P* values were greater than 0.05 (Table 1). The results indicated that Fusarium wilt resistance in CW9-8 should be controlled by a single dominant gene, and named *PsFwC9*.

**Table 1:** Genetic segregation in response to *F. oxysporum* f. sp. *pisi* in the F_2_ population derived from Chengwan 9-1 and Chengwan 9-8.

Parent and the cross	Generation	Amount	Observed number^a^		Chi-squared tests^b^		
			R	S	Expected ratio	*χ* ^2^	*P*
Chengwan 9-8 (CW9-8)	P_1_	30	30				
Chengwan 9-1 (CW9-1)	P_2_	30		30			
CW9-1 × CW9-8-A	F_2_	98	68	30	3:1	1.65	.20
CW9-1 × CW9-8-B	F_2_	72	53	19	3:1	0.07	.79
CW9-1 × CW9-8-C	F_2_	54	38	16	3:1	0.62	.43
CW9-1 × CW9-8-D	F_2_	72	58	14	3:1	1.19	.28
CW9-1 × CW9-8-E	F_2_	73	53	20	3:1	0.75	.39
CW9-1 × CW9-8-F	F_2_	46	32	14	3:1	0.72	.39
CW9-1 × CW9-8-G	F_2_	45	34	11	3:1	0.01	.93
CW9-1 × CW9-8-H	F_2_	55	39	16	3:1	0.49	.48
CW9-1 × CW9-8-I	F_2_	29	21	8	3:1	0.10	.74
CW9-1 × CW9-8	F_2_	544	396	148	3:1	1.78	.18

a R, resistant; S, susceptible.

b Expected ratio: dominant trait individual/recessive trait individual = 3:1. The *χ*^2^ < 3.84 and *P* > 0.05 indicated that the theoretical segregation ratio is significantly correlated with the actual.

Furthermore, among the 13 other pure lines of the CW9, five lines showed resistance, whereas eight lines were susceptible or highly susceptible (Table S1). Leaf samples from the five resistant and eight susceptible individuals were collected for further haplotype analysis.

### Reads mapping and BSA-seq analysis

Utilizing the Illumina platform and fastp v0.23.1 software, ~48.30, 48.89, 92.22 and 89.91 Gb clean data were generated for the CW9-1, CW9-8, CW9S and CW9R, respectively. Following the alignment of clean data to the reference genome Caméor v1a, a total of 320 million and 324 million paired-end 150-bp mapped reads were obtained for parents CW9-1 and CW9-8, achieving mean depths of 14.81× and 14.98×, with 4× genome coverage rates of 74.04% and 74.19%, respectively (Table 2). For the two bulks CW9S and CW9R, 614 million and 599 million paired-end 150-bp mapped reads were captured, resulting in mean depths of 25.78× and 25.12×, and 4× genome coverage rates of 78.52% and 78.11%, respectively (Table 2).

**Table 2:** WGRS of parents and bulks using Illumina sequencing.

Genotype sample	Clean base (Gb)	Number of clean reads	Mapping rate (%)	Mean depth (×)	4× genome coverage rate (%)
Chengwan 9-1	48.30	320 074 459	99.39	14.81	74.04
Chengwan 9-8	48.89	324 043 762	99.41	14.98	74.19
CW9S	92.22	610 790 390	99.34	25.78	78.52
CW9R	89.91	599 377 398	99.44	25.12	78.11

A total of 23 062 829 variant loci were detected on the seven major chromosomes of pea through comparison with the reference genome, yielding 1 713 204 reliable SNPs and InDels following continuous filtering (Table S4). In this study, InDels were regarded as SNPs to calculate and analyze the SNP index. The SNP index and ΔSNP index value were calculated for each SNP locus to determine the genomic region associated with *Fop* resistance. A 201.5-Mb genomic region (243 351 840–444 788 063) on chr4LG4, showing a high average ΔSNP index, was speculated to be the candidate region containing *PsFwC9* (Fig. 1B). Within this extensive candidate region, two specific areas, measuring 3.88 Mb (307 291 808–311 172 916) and 3.74 Mb (424 037 046–427 779 104), exhibited an average SNP index value greater than 0.95 for CW9R and less than 0.5 for CW9S, with ΔSNP index values exceeding 0.9, leading to the conclusion that these locations were strongly associated with *PsFwC9* (Fig. 1B).

### Fine mapping of *PsFwC9*

According to the BSA-seq analysis and gene annotation, 40 KASP markers (Table S2) were first developed within the 201.5 Mb candidate region on chr4LG4 and used to genotype 98 individuals from the CW9-1 × CW9-8-A F₂ population. Six markers showed consistent linkage to *PsFwC9*, with A016310, A016313 and A016467 found in the 3.88 Mb (307.29–311.17 Mb) highly correlated region, while A016507, A016510 and A016511 were situated in the 3.74 Mb (424.04–427.78 Mb) closely linked region, with the two intervals separated by ~112.87 Mb (Fig. 1C). To define the candidate region of *PsFwC9*, 20 additional KASP markers (Table S2) were developed to genotype 369 individuals from five F₂ populations (A–E) derived from CW9-1 × CW9-8. The *PsFwC9* was delimited to an 834.79-kb interval between markers A016664 and A016511, co-segregating with markers A016665, A016508, A016510 and A016615, located within the 424.04- to 427.78-Mb candidate region (Fig. 1D). Subsequently, to further refine the candidate region, the mapping population was expanded to nine F_2_ populations (A–I) of CW9-1 × CW9-8, comprising 544 individuals, and 10 more KASP markers were developed for detection within this interval. The candidate region of *PsFwC9* was narrowed down to 817.06 kb, flanked by markers A016508 and A016511, and the *PsFwC9* was co-segregated with markers A016510, A016615, A016836 and A016837, indicating that no recombination events occurred (Fig. 1E). Moreover, the 544 F_2_ individuals could be categorized into 10 haplotypes based on the 10 markers. The presence of SNPs corresponding to markers A016510, A016615, A016836 and A016837 in individuals reliably demonstrated resistance to Fusarium wilt, whereas individuals without these four SNPs presented a susceptible phenotype (Fig. 1F).

### Haplotype analysis and diagnostic marker of *PsFwC9*

The six KASP markers, either co-segregated or tightly linked with the *PsFwC9*, were utilized to detect 218 pea accessions with known resistance phenotypes, aiming to determine SNPs related to Fusarium wilt resistance and to discover accessions carrying the unique *PsFwC9* haplotype. A total of 10 haplotypes were identified among 218 accessions, where accessions with SNP variation at the marker A016615 consistently exhibited resistance to Fusarium wilt, while those without this variation showed either resistant or susceptible phenotypes (Fig. 1G). Some accessions containing the other variations of five markers exhibited susceptibility to Fusarium wilt, indicating that these SNPs are not associated with the *PsFwC9* resistance gene.

These findings suggest that the ‘A’ to ‘G’ SNP variation at marker A016615 could be a crucial factor in determining pea resistance to Fusarium wilt (Fig. 1H). Although the variation at this marker locus was detected in only 13 accessions (Table S1), among which six pure lines derived from CW9 were homozygous for the mutation (G/G) and the remaining seven accessions (including CW9 itself) were heterozygous (G/A), the marker A016615 successfully identified the resistance-associated allele in accessions and populations with distinct genetic backgrounds, demonstrating strong specificity and stability across diverse accessions. Therefore, the KASP marker A016615 was designated as the diagnostic molecular marker for the *PsFwC9* resistance gene.

### Candidate gene identification of *PsFwC9*

**Figure 2:**
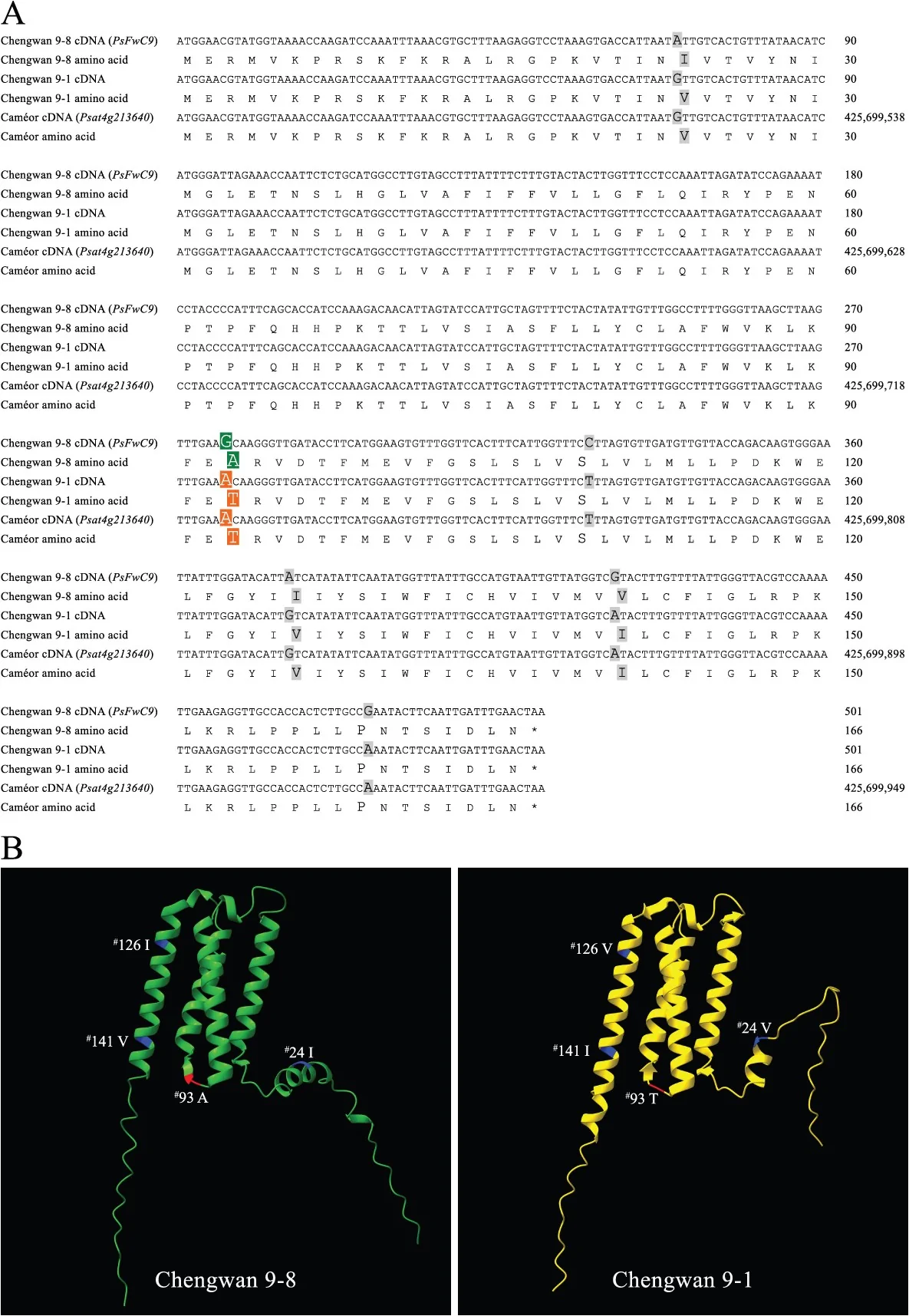
Comparison of DNA and amino acid sequences (A) and predicted protein structures (B) of the candidate gene *Psat4g213640* between Chengwan 9-8 and Chengwan 9-1. (A) The cDNA sequence of the candidate gene is completely consistent with the corresponding genomic DNA sequence in the reference genome. (B) The numbers indicate amino acid positions, and single-letter codes represent amino acid residues.

Haplotype analysis revealed that marker A016615 was the only locus specifically associated with the resistance gene *PsFwC9*. Based on the Caméor reference genome and sequence alignment, the functional SNP, located at 425 699 725 bp on chr4LG4, corresponding to position 277 bp within *Psat4g213640*, involved a ‘A/G’ substitution, leading to an amino acid change from threonine (Thr) to alanine (Ala). Moreover, five additional mutation loci were identified in the *Psat4g213640* between the resistant parent CW9-8 and the susceptible parent CW9-1. Among them, two were synonymous substitutions, while the remaining three corresponded to the co-segregated markers within the gene, resulting in only conservative amino acid changes between valine (Val) and isoleucine (Ile) [30, 31] (Fig. 2A). These results indicate that A016615 was the only disease-resistance-associated locus and *Psat4g213640* could be the candidate gene of *PsFwC9*.

The predicted protein structures of Psat4g213640^CW9-8^ and Psat4g213640^CW9-1^ exhibited a similar overall fold, both consisting of three α-helices in the N-terminal region followed by an extended C-terminal coil (Fig. 2B). Nevertheless, subtle conformational distinctions were observed. The C-terminal region of Psat4g213640^CW9-1^ appeared slightly more extended, whereas Psat4g213640^CW9-8^ displayed a more compact and inward-bent configuration. Moreover, four amino acid substitutions were identified between the two proteins: Val-24-Ile, Thr-93-Ala, Val-126-Ile, and Ile-141-Val. Among them, theThr-93-Ala replacement, located near the loop connecting α-helices, results in the loss of a hydroxyl group and potential hydrogen bonds, likely affecting local structural stability or interactions with other membrane-associated components. The remaining substitutions represented conservative hydrophobic exchanges that might have slightly helix packing but were unlikely to cause major structural rearrangements.

### Expression patterns of *PsFwC9*

**Figure 3:**
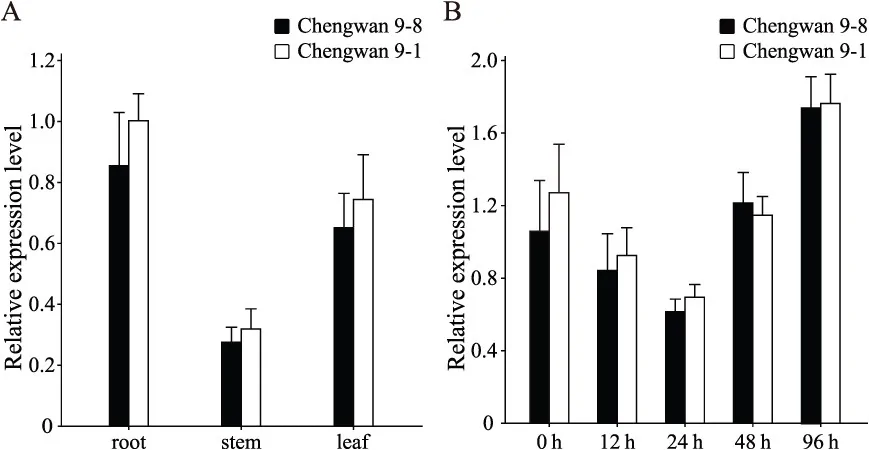
Expression patterns of *PsFwC9* candidate gene *Psat4g213640*. (A) Expression patterns of *Psat4g213640* in roots, stems and leaves of Chengwan 9-8 and Chengwan 9-1. (B) Relative expression levels of *Psat4g213640* in the roots of Chengwan 9-8 and Chengwan 9-1 at 0, 12, 24, 48 and 96 h after inoculation with *F. oxysporum* f. sp. *pisi*. In (A) and (B), the qRT-PCR results were normalized to the expression of reference gene *PsActin*, and values were presented as the mean (±standard error) of three independent replicates.

In order to verify the correlation between the candidate gene and Fusarium wilt resistance, qRT-PCR analysis was conducted to examine the expression pattern of *Psat4g213640* in the susceptible parent CW9-1 and the resistant parent CW9-8. The expression levels of *Psat4g213640* were examined by qRT-PCR in the roots, stems and leaves of CW9-8 and CW9-1 at the seedling stage. Specifically, the *Psat4g213640* exhibited the highest expression in roots and the lowest in stems in both parents, but no significant difference in expression levels was observed between CW9-8 and CW9-1 in any of the tested tissues (Fig. 3A).

Subsequently, the expression of *Psat4g213640* in the roots of pea seedlings was quantitatively analyzed at five different time points after inoculation. The results showed that the expression patterns of *Psat4g213640* were similar between the two parental lines. Both parental lines exhibited a similar temporal expression pattern of *Psat4g213640* after inoculation, with transcript levels decreasing initially and reaching the lowest point at 24 h, followed by a gradual increase that peaked at 96 h. No significant differences in *Psat4g213640* expression were observed between the two parents at any of the tested time points (Fig. 3B). Therefore, these results suggested that the Fusarium wilt resistance in CW9-8 might not be associated with the expression level of the candidate gene *Psat4g213640*.

**Figure 4:**
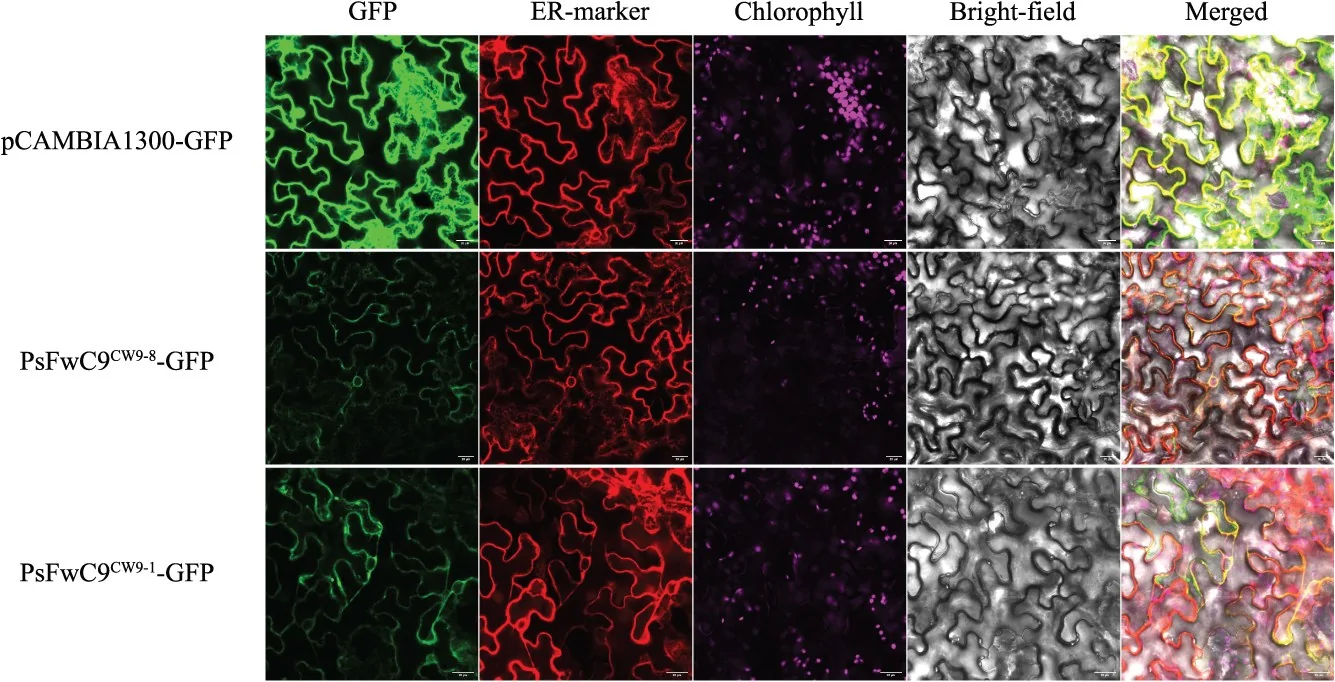
Subcellular co-localization of *PsFwC9* candidate gene *Psat4g213640* in *N. benthamiana* leaves.

To determine the subcellular localization of the *PsFwC9* candidate gene, the coding sequence of *Psat4g213640* was fused to GFP to generate the PsFwC9^CW9-8^-GFP and PsFwC9^CW9-1^-GFP constructs. The recombinant vector was transiently expressed in epidermal cells of *Nicotiana benthamiana* via *Agrobacterium tumefaciens* strain GV3101. Confocal microscopy showed that the control GFP signal was distributed throughout the cytoplasm and nucleus, whereas the PsFwC9^CW9-8^-GFP and PsFwC9^CW9-1^-GFP fluorescence displayed a typical reticular pattern and was mainly observed in the endoplasmic reticulum (ER), with weak signals detected at the plasma membrane (Fig. S1). Co-localization analysis with an ER marker showed that the GFP signal overlapped strongly with the ER marker, confirming that *PsFwC9* is localized to the endoplasmic reticulum (Fig. 4).

### Functional validation of *Psat4g213640* in Fusarium wilt resistance

Due to the lack of an efficient genetic transformation system in pea, a hairy root system was used to investigate the role of the *PsFwC9* candidate gene *Psat4g213640^CW9-8^* in Fusarium wilt resistance. The overexpression construct *PsFwC9*-OE was introduced into *Agrobacterium rhizogenes* strain K599 to induce hairy roots in the susceptible parent CW9-1. Vigorously growing hairy roots were selected for expression analysis and Periodic acid–Schiff (PAS) staining.

**Figure 5:**
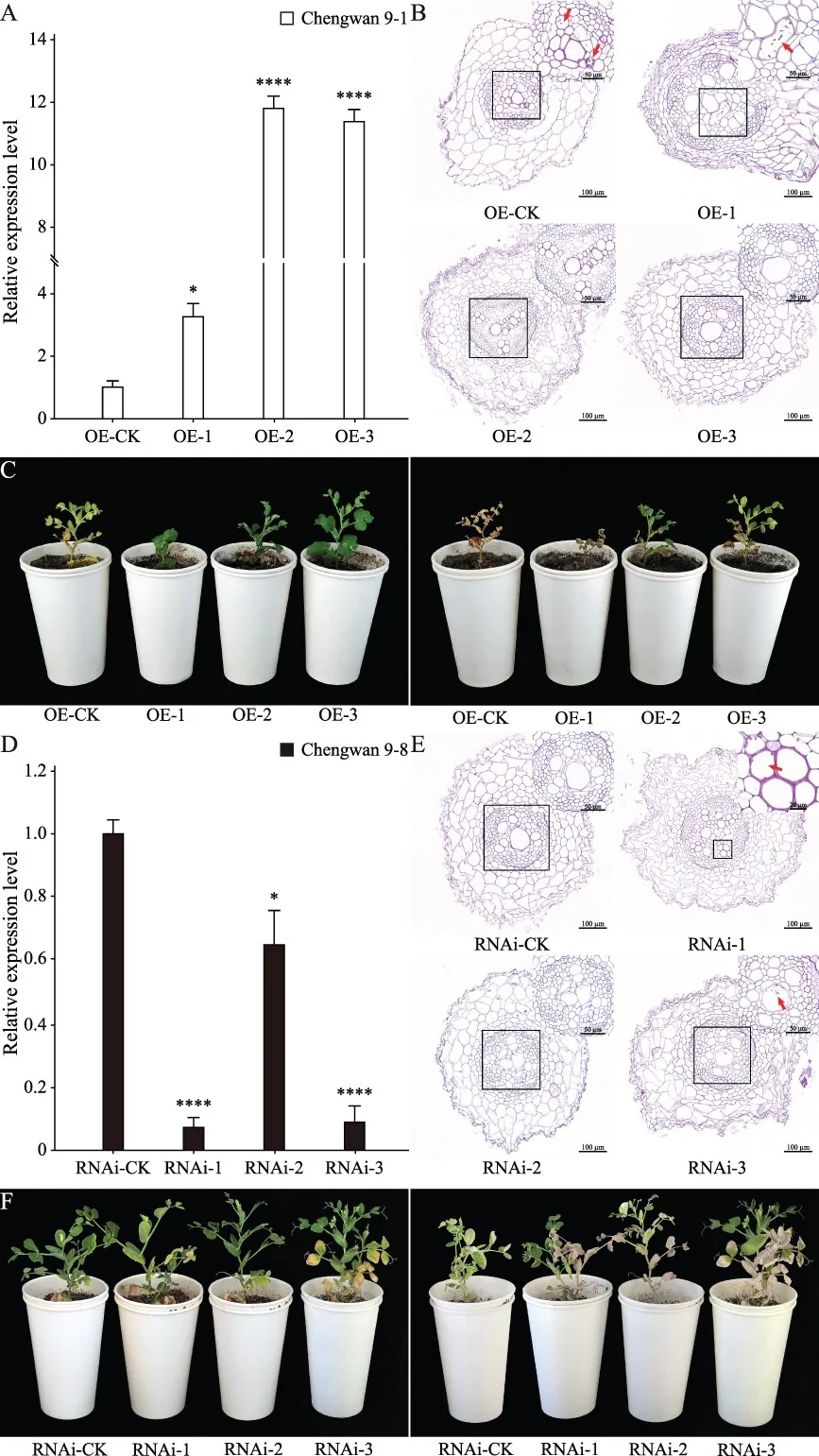
Overexpression and RNA interference of *PsFwC9* candidate gene *Psat4g213640* in hairy roots. (A) and (D) showed the expression levels of *Psat4g213640* in the best growing hairy root of overexpression and RNA interference, respectively. The qRT-PCR results were normalized to the expression of reference gene *PsActin*, and values were presented as the mean (±standard error) of three independent replicates. The symbols ‘^*^’ and ‘^****^’ indicate significant differences at the 0.05 and 0.0001 levels, respectively. (B) and (E) were the PAS staining results of the vigorously growing hairy root of overexpression and RNA interference, respectively. (C) and (F) were the phenotypic evaluation of overexpression and RNA interference at different times after inoculation, respectively.

The qRT-PCR analysis revealed that the expression levels of *Psat4g213640* in the hairy roots of the overexpression lines CW9-1_OE-1, CW9-1_OE-2 and CW9-1_OE-3 were significantly higher than those in the control, with expression levels increased by 3.2-, 11.8- and 11.3-fold, respectively. The differences were statistically significant (*P* < 0.05) and highly significant (*P* < 0.00001) (Fig. 5A). PAS staining of the overexpression hairy roots showed that at 24 h post-inoculation, *Fop* microconidia had invaded the vascular tissues of both the control and CW9-1_OE-1 roots, whereas no *Fop* microconidia were observed in the vascular tissues of CW9-1_OE-2 and CW9-1_OE-3 roots (Fig. 5B). The visible disease symptoms appeared in the control plants at 10 dpi, whereas no obvious symptoms were observed in CW9-1_OE-1, CW9-1_OE-2 or CW9-1_OE-3 plants. At 18 dpi, both the control and CW9-1_OE-1 plants had completely wilted, whereas CW9-1_OE-2 and CW9-1_OE-3 plants exhibited only mild wilting symptoms and survived until ~30 dpi, when complete wilting eventually occurred (Fig. 5C). These results indicated that overexpression of *Psat4g213640* enhanced resistance to Fusarium wilt, effectively delaying the onset of disease symptoms and extending seedling survival.

RNA interference (RNAi) analysis was subsequently performed in the resistant parent CW9-8 using the same hairy root system. The qRT-PCR results showed that the expression levels of *Psat4g213640^CW9-8^* in the hairy roots of the RNAi lines CW9-8_RNAi-1, CW9-8_RNAi-2 and CW9-8_RNAi-3 were reduced to 7.3%, 64.9% and 8.9% of the control levels, respectively, showing highly significant (*P* < 0.0001) and significant (*P* < 0.05) differences (Fig. 5D). PAS staining at 24 h post-inoculation revealed that a small number of *Fop* microconidia had invaded the vascular tissues of CW9-8_RNAi-1 and CW9-8_RNAi-3 roots, whereas no fungal spores were observed in the control or CW9-8_RNAi-2 roots (Fig. 5E). At 18 dpi, CW9-8_RNAi-1 and CW9-8_RNAi-3 plants showed early wilting symptoms, characterized by leaf chlorosis, whereas no visible symptoms were observed in the control or CW9-8_RNAi-2 plants. At 40 dpi, all plants remained alive; however, one branch of both CW9-8_RNAi-1 and CW9-8_RNAi-3 had completely wilted, and approximately half of the leaves on CW9-8_RNAi-2 displayed chlorosis and wilting symptoms. The disease index of CW9-8_RNAi plants increased significantly, while the control plants showed only natural senescence of basal leaves (Fig. 5F). These results indicated that RNAi-mediated silencing of *Psat4g213640* compromised resistance to Fusarium wilt.

Taken together, these results demonstrate that *Psat4g213640* played a positive role in resistance to Fusarium wilt in pea.

## Discussion

The pea Fusarium wilt, caused by *Fop*, is a destructive vascular disease that results in severe yield losses and continues to be a major constraint to pea production worldwide [9–11]. The deployment of resistant cultivars is widely recognized as the most economical, efficient and environmentally sustainable strategy for controlling Fusarium wilt [10, 11, 15]. Therefore, identifying Fusarium wilt resistance genes in pea and clarifying their genetic characteristics are essential for the innovation, utilization and molecular breeding of resistant cultivars [16–18]. However, *F. oxysporum* can gain virulence to overcome single-gene resistance through mutation or horizontal gene transfer, suggesting that breeding strategies relying solely on individual resistance genes carry potential risks [32–35]. The identification and functional validation of new resistance genes are vital for enriching resistance resources and addressing these challenges, providing more genetic options for pea breeding and enabling gene pyramiding strategies to enhance resistance durability and reduce the risk posed by pathogen variability [36, 37]. Therefore, the CW9-8, an elite pea pure line carrying new resistance gene to Fusarium wilt, was selected for fine mapping and functional characterization of the resistance gene, aiming to provide a novel gene and diagnostic marker for Fusarium wilt resistance breeding in pea, as well as new insights into the underlying mechanisms of resistance.

The BSA method is an effective method for rapidly identifying trait-associated markers by constructing DNA bulks from individuals displaying extreme phenotypes [38]. With the advancement of next-generation sequencing technologies and the release of reference genomes for multiple crops, it is now possible to efficiently detect high-throughput SNPs and InDels related to target traits by combining whole genome resequencing with BSA [39, 40]. Peas have a significantly lower propagation rate compared to gramineous crops like rice, wheat and maize, making it difficult to fine-map target genes using traditional mapping techniques [41–43]. Unlike traditional methods that require large genetic populations, the BSA-seq technique can efficiently identify candidate regions and molecular markers associated with target traits by sequencing and analyzing bulks from a limited number of extreme individuals, and this approach has been successfully utilized for identifying many traits in pea, such as disease resistance [16, 44], stress tolerance [45] and morphological traits [46, 47]. In this study, the candidate region associated with resistance to Fusarium wilt was identified using the BSA-seq technique. Following this, the resistance gene *PsFwC9* was finely mapped using traditional linkage analysis, along with an expanded mapping population, which narrowed the candidate region to 817.06 kb with four co-segregating markers. The strategy of combining BSA-seq with traditional mapping methods accelerated the identification of the *PsFwC9* candidate gene, which could be utilized for gene mapping of target traits in peas and other crops as well.

Previous studies have reported several Fusarium wilt resistance genes in pea, including *Fw* on chr5LG3 conferring resistance to *Fop* race 1 [17, 19], *Fwf* on chr6LG2 providing resistance to race 5 [20, 22] and *Fnw4*, a major-effect gene on chr4LG4 associated with resistance to race 2 [21]. More recently, the resistance gene *FwS1* was identified on chr6LG2 within a 91.4-kb region and was proposed to confer resistance to race 5, with genetic evidence suggesting that *FwS1* may be closely linked to, or even identical with, the previously reported *Fwf* locus [16]. In this study, haplotype analysis revealed that only marker A016615 was associated with Fusarium wilt resistance, and the locus corresponding to this marker was identified as 277 bp of the gene *Psat4g213640* on chr4LG4 in the pea reference genome. Sequence comparison between the resistant parent CW9-8 and the susceptible parent CW9-1 revealed six SNP variations in *Psat4g213640*. Among them, the SNP corresponding to marker A016615 involved an A-to-G substitution, resulting in an amino acid change from Thr (a polar amino acid) to Ala (a nonpolar amino acid). The remaining five variations consisted of conservative substitutions between Val and Ile or synonymous mutations, which were less likely to substantially affect protein function [30, 31]. Notably, the candidate gene *Psat4g213640* differed from the previously reported *FwS1* candidate gene *Psat6g003960* located on chr6LG2. Sequence analysis of *Psat6g003960* revealed the presence of a typical NB-ARC domain, a conserved functional ATPase module widely found in classical plant resistance proteins and known to regulate resistance-protein activation. In contrast, *Psat4g213640* lacked recognizable resistance-related domains and showed no clear functional annotation in the Caméor v1a and Zhongwan 6 v1.0 reference genomes or in public databases such as NCBI (https://www.ncbi.nlm.nih.gov/) or TAIR (https://www.arabidopsis.org/). The structural and genomic differences between these genes suggested that *PsFwC9* might represent a distinct resistance locus from the previously reported *FwS1* or *Fwf* region and might contribute to Fusarium wilt resistance through a mechanism different from classical NB-ARC type resistance proteins. Based on the combined evidence from fine mapping, haplotype analysis and sequence comparison, *Psat4g213640* was considered the candidate gene underlying *PsFwC9*. In addition, the SNP represented by marker A016615 could serve as a diagnostic marker for the precise identification of the *PsFwC9* allele in different pea populations and germplasm resources. To our knowledge, this study provides the first evidence of a Fusarium wilt resistance gene candidate located on pea chr4LG4.

Gene expression analysis is a commonly used approach to verify target gene function. Among these methods, qRT-PCR has become one of the most widely applied techniques for measuring gene expression levels owing to its superior accuracy and sensitivity [48–50]. In qRT-PCR, gene expression quantification can be performed using either absolute or relative quantification methods [49, 51, 52]. Since absolute quantification requires converting data into absolute copy numbers, a process that is often unnecessary in practical research [51], relative quantification has become the more widely adopted approach due to its simplicity and practicality [49, 50, 52]. In this study, the expression of the candidate gene *Psat4g213640* was quantitatively analyzed using qRT-PCR. The results showed that *Psat4g213640* was expressed in the roots, stems and leaves of both parents, with the highest expression in roots. After *Fop* inoculation, the expression patterns of *Psat4g213640* in both parents exhibited similar trends and no significant differences were detected, suggesting that the resistance of CW9-8 might not be directly linked to the expression level of *Psat4g213640*, but rather was conferred by alterations in its protein structure or function, since amino acid substitutions could modify the protein ability to interact with pathogen effectors or activate downstream defense signaling pathways, thereby influencing the plant resistance to disease [53–56].

The *A. rhizogenes*-mediated hairy root transformation provides a powerful transient gene expression system for studying root biology and has been successfully applied to more than 100 plant species, including soybean and common bean [50, 57–59]. This technique enables researchers to rapidly obtain transgenic hairy roots, allowing efficient functional characterization of genes, particularly those involved in root development, nutrient uptake and stress responses. Moreover, the hairy root system circumvents the complexity and time consumption associated with conventional transgenic technologies, offering a practical platform for root-specific gene function analysis in crops that lack an established stable transformation system [57, 59]. Gene overexpression and gene silencing are two complementary strategies for functional gene validation. Overexpression enhances the expression level of the target gene to assess the potential gain of function under elevated expression, whereas gene silencing reduces gene expression to investigate the phenotypic or functional consequences of expression suppression [50, 53]. In this study, the hairy root system was employed to perform overexpression and gene silencing experiments for the candidate gene *Psat4g213640*. Overexpression of *Psat4g213640^CW9-8^* significantly enhanced the resistance of the susceptible parent CW9-1 to Fusarium wilt, delaying disease onset and progression, indicating that this gene conferred resistance in CW9-1. In contrast, RNAi of *Psat4g213640^CW9-8^* markedly reduced the resistance of the resistant parent CW9-8, further confirming that *Psat4g213640^CW9-8^* is a key gene underlying Fusarium wilt resistance in CW9-8. It should be noted that due to the presence of untransformed roots or insufficient efficiency of overexpression and silencing in *A. rhizogenes*-induced hairy root systems, the expression levels of some transformed roots showed no significant differences from the controls, leading the overall plant resistance to gradually approach its original phenotype [50, 53, 57, 59]. Nevertheless, this study not only verified the critical role of *Psat4g213640^CW9-8^* in Fusarium wilt resistance but also demonstrated that the hairy root system served as a reliable tool for investigating root-associated gene functions. Future studies could improve the accuracy and reproducibility of this approach by optimizing transformation efficiency and screening strategies.

Protein structure prediction serves as a fundamental basis for elucidating biological function [50, 53, 55]. In this study, the predicted structural comparison revealed that *Psat4g213640^CW9-1^* and *Psat4g213640^CW9-8^* shared a similar overall fold but exhibited subtle differences, including a slightly more extended C-terminal region in *Psat4g213640^CW9-1^* and four amino acid substitutions (Val-24-Ile, Thr-93-Ala, Val-126-Ile, and Ile-141-Val). Among these, the Thr-93-Ala substitution located near the loop connecting α-helices might influence local structural stability and flexibility, thereby affecting protein–protein interactions. Threonine residues contain a hydroxyl group capable of participating in hydrogen bonding and phosphorylation-related interactions, whereas substitution with alanine removes this functional group and may disrupt local stabilizing interactions within α-helices or adjacent structural elements [60–62]. Similar Thr-to-Ala substitutions have been shown to alter protein stability or conformational dynamics by modifying hydrogen-bond networks or helix capping interactions. For example, mutation of Thr-101Ala in the nitrate transporter NRT1.1 has been demonstrated to change its functional state and transport activity through phosphorylation-dependent structural regulation [62, 63]. In addition, structural studies of proteins such as thioredoxin have indicated that Thr-to-Ala substitutions can weaken helix stability and alter packing interactions, thereby affecting protein conformational flexibility [60, 64]. These observations suggest that the amino acid substitutions identified in *Psat4g213640*, particularly the Thr-to-Ala change, may influence local structural stability or protein interactions, which could ultimately contribute to the functional differences observed between resistant and susceptible lines. Further biochemical and structural studies will be required to determine how the amino acid substitutions influence the structure and activity of *PsFwC9*.

Subcellular localization analysis is a crucial step for further elucidating protein function, as the intracellular localization of a protein is closely associated with its biological role [53, 55]. In this study, subcellular localization analysis showed that the protein encoded by *Psat4g213640* was predominantly localized to the endoplasmic reticulum, suggesting that it may participate in plant defense responses through ER-associated signaling regulation or protein processing. The endoplasmic reticulum is an important cellular compartment responsible for protein synthesis, folding and trafficking, and it plays a crucial role in plant immune responses. Many immune-related proteins undergo folding and maturation in the endoplasmic reticulum before being transported to the plasma membrane or other cellular compartments, where they participate in pathogen recognition and signal transduction [65–67]. In addition, the endoplasmic reticulum is closely associated with intracellular Ca^2+^ signaling, reactive oxygen species production, and stress responses [68–71]. Therefore, the ER-localized *PsFwC9* may contribute to resistance against Fusarium wilt in pea by regulating the processing and transport of immune-related proteins or by participating in ER-associated defense signaling pathways. Future studies could further explore the functional mechanisms of *Psat4g213640*^CW9-8^ by combining transient and stable transformation systems, transcriptomic profiling and protein to protein interaction assays to validate its role in membrane-associated defense signaling.

## Conclusion

In summary, this study provided significant insights into the genetic basis of Fusarium wilt resistance in pea. A novel Fusarium wilt resistance gene, *PsFwC9*, in pea line CW9-8 was finely mapped through BSA-seq analysis, and candidate gene *Psat4g213640* was identified by haplotype analysis and gene cloning. The qRT-PCR analysis showed that *Psat4g213640* was expressed in multiple tissues of both resistant and susceptible parents, but its expression level was not significantly correlated with resistance. Protein structure prediction revealed that the amino acid substitution in *Psat4g213640* altered the overall conformation of the protein. Subcellular co-localization confirmed that the *Psat4g213640*-encoded protein was localized to the endoplasmic reticulum. Functional validation using the hairy root transformation system demonstrated that overexpression of *Psat4g213640^CW9-8^* significantly enhanced resistance to Fusarium wilt in the susceptible line CW9-1, whereas RNAi-mediated silencing of *Psat4g213640^CW9-8^* in the resistant line CW9-8 markedly reduced its resistance. Collectively, these findings indicate that *PsFwC9* is a key gene conferring Fusarium wilt resistance in pea, and the locus and molecular markers identified herein provide a solid foundation for future molecular breeding and genetic improvement of Fusarium wilt resistance in pea.

## Materials and methods

### Plant materials and pathogen inoculum

The heterozygous resistant cultivar CW9 was acquired from SAAS in 2019. Then, 15 pure lines were developed from CW9 through continuous single plant selection and Fusarium wilt identification for three consecutive years. Among these, the purified resistant line CW9-8 was used as the paternal parent, and the purified susceptible line CW9-1 as the maternal parent, to construct an F_2_ population for BSA-seq analysis and fine mapping. Moreover, seven differential cultivars for race identification of *Fop* (Litter Marvel, Darkskin Perfection, New Era, New Season, WSU 23, WSU 28 and WSU 31) obtained from the United States Department of Agriculture, Agricultural Research Service (USDA ARS) were used as controls. The 196 accessions with known resistance responses from the China National Crop Genebank were employed for haplotype analysis (Table S1) [16].

The *Fop* race 5 isolate PF22b, obtained from Jianyang City, Sichuan Province, was employed for phenotypic evaluations [10]. This isolate was stored at −80°C for long-term preservation and cultured on potato dextrose agar (PDA) medium at 27°C for experimental use.

### Phenotypic evaluation for Fusarium wilt

Nine F₂ populations derived from CW9-1 × CW9-8, consisting of 544 individual seeds, were planted sequentially in paper cups (600 ml) filled with fresh vermiculite. Approximately 150 plants and 30 parental lines were grown in each sowing batch. Each paper cup was planted with five seeds, and the cups were placed in greenhouses at 24°C–26°C for 2 weeks. The seedlings were inoculated with *Fop* race 5 isolate PF22b using a modified root-dipping inoculation method described by Bani *et al.* [15] and Deng *et al.* [10, 16]. Briefly, small mycelial plugs were cut from the edges of 7-day-old fungal colonies and placed into pea broth medium, then shaken at 120 rpm and 27°C for 2 days. After filtration, the microconidia suspension was adjusted to a final concentration of 1.0 × 10^7^ microconidia/ml. Seedlings were uprooted from vermiculite and washed, and the bottom third of the roots was trimmed. The remaining roots were dipped in the microconidia suspension for 3 min before being transplanted into new paper cups. The control seedlings underwent the same process, with sterile water substituted for the microconidia suspension. The inoculated seedlings were grown under natural light in greenhouses at 27°C for an additional 28 days, with watering as needed. Disease severity was assessed based on the percentage of symptomatic leaves (PSL) for each individual seedling following the method of Deng *et al.* [10, 16], where the PSL between 0 and 50 indicated the seedlings were resistant, while values between 50 and 100 signified susceptible.

### Construction of extreme bulks and Illumina sequencing

Leaf samples of both parental lines and individual plants were collected from 2 days post-inoculation (dpi) seedlings, and their genomic DNA was extracted using the DNAsecure Plant Kit DP320-03 (Tiangen Biotech, Beijing, China), according to the manufacturer’s instructions. The DNA quality and concentration of each sample were detected using 1% agarose gel electrophoresis and a NanoDrop 2000/2000c spectrophotometer (Thermo Fisher Scientific, Waltham, MA, USA), respectively. Following phenotypic evaluation, the DNA samples from 15 extreme resistant and 15 extreme susceptible seedlings in CW9-1 × CW9-8-A F_2_ population were combined to create a resistant bulk (CW9R) and a susceptible bulk (CW9S), respectively. The two bulks, CW9R and CW9S, along with the parents, CW9-8 and CW9-1, were sent to Novogene (Tianjin, China) for construction of Illumina libraries and whole-genome resequencing (WGRS), with the bulks sequenced at 20× coverage, and the parents at 10× coverage.

### Identification of genomic region responsible for Fusarium wilt resistance

The clean data reads were obtained after removing paired-end reads with low-quality (*Q* < 15), low-length reads (<20 bp), more than five undetermined bases and adapter sequences in raw data reads by fastp v0.23.4 software [72]. The high-quality reads were aligned to the pea reference genome Caméor v1a (https://urgi.versailles.inra.fr/Species/Pisum) using bwa-mem2 v2.2.1 software [73, 74]. The alignment results were then sorted and filtered for accurate reads using samtools v1.18 software [75], while GATK v4.4.0 [76] was applied to mark and remove duplicate reads, as well as to calculate the mapping rate, 4× genome coverage and mean depth. The SNPs and InDels were detected using GATK HaplotypeCaller [76, 77], vcftools v0.1.16 software [78] and Linux commands, based on the following criteria: (1) variations present in all samples; (2) homozygous variations in parental samples; and (3) variant loci on major chromosomes with sequencing depth ≥4×.

The high-quality differential SNPs and InDel sites were identified following the filtering process. The SNP index, defined as the ratio of reads containing SNPs and InDels to the total number of reads, was calculated for the CW9R and CW9S [39]. The ΔSNP index, representing the difference in SNP index values between the two bulks, was used to identify candidate genomic regions associated with disease resistance. Each locus in the ΔSNP index was calculated by subtracting the SNP index of the CW9R from that of the CW9S, following this formula:


$$ \Delta \mathrm{SNP}\ \mathrm{index}=\frac{\mathrm{AltR}}{\mathrm{AltR}+\mathrm{RefR}}-\frac{\mathrm{AltS}}{\mathrm{AltS}+\mathrm{RefS}} $$

where R = the CW9R, S = the CW9S, Ref = the read depth matching that of the reference genome, and Alt = the read depth deviating from that of the reference genome. The values of two SNP indexes and ΔSNP index were visualized with QTL-seq v2.2.3 software [40] and Adobe Illustrator 2025 (http://www.adobe.com). A higher ΔSNP index indicates a stronger likelihood that the SNPs and InDels were involved in *Fop* resistance or were linked to a gene regulating this trait.

In defining the candidate region, the absence of QTLs at that position was assumed, resulting in the random allocation of locus genotyping between the two progenies during genetic evaluation. The SNPs and InDels with ΔSNP index ≥0.75 were selected for annotation with SnpEff v5.2 software [79]. The available genomic annotation files (gff/gtf, https://urgi.versailles.inra.fr/Species/Pisum) were utilized to determine the genomic locations and mutation types of SNPs and InDels.

### Marker development and fine mapping

The nonsignificant SNP and InDel loci were filtered out according to the gene annotation, and the retained loci with 200-bp flanking sequences on both sides were extracted using TBtools-II v2.027 software [80]. A local BLAST was then conducted to align these extracted sequences with the reference genome Caméor v1a, excluding sequences with more than four copies in the genome. The SNP and InDel loci within the target genomic region were chosen for conversion into Kompetitive Allele-Specific PCR (KASP) markers using the Polymarker online platform (http://www.polymarker.info/). All KASP primers and subsequent primers were synthesized by Sangon Biotech (Shanghai, China).

The 5′ ends of both forward primers in each designed KASP assay was modified by adding the standard FAM-label or HEX-label fluorescent sequence (FAM tail: 5′-GAAGGTGACCAAGTTCATGCT-3′; HEX tail: 5′-GAAGGTCGGAGTCAACGGATT-3′) (Table S2). The amplification of KASP markers was performed on a Douglas Scientific Array Tape Platform (China Golden Marker, Beijing) in a 1.6-μl reaction volume, containing 0.8-μl DNA (~22 ng/μl), 0.022-μl primer mix, 0.4-μl KASP V4.0 2× Mastermix 1536 (LGC Biosearch Technologies, Shanghai, China) and 0.4-μl ddH_2_O. PCR amplification was conducted on a Soellex PCR Thermal Cycler (Douglas Scientific, Alexandria, MN, USA) with the following protocol: an initial denaturation at 94°C for 15 min, followed by 10 touchdown cycles of 94°C for 20 s and 65°C for 1 min (decreasing by 0.6°C each cycle), then 26 cycles of 94°C for 20 s and 55°C for 1 min, concluding with a final cooling step at 4°C. Fluorescent end-point readings were performed using the Araya fluorescence detection system (integrated with the Douglas Scientific Array Tape Platform), and genotypes and clusters were visualized with Kraken2 v2.0.8 software [81]. Sequential evaluation of the F_2_ populations was conducted using KASP markers. Candidate genes were identified based on co-segregating markers, while markers with more than 5% recombination events in the same population would be removed. Then, the physical maps of KASP markers linked to the Fusarium wilt resistance gene were generated through Adobe Illustrator 2025.

### Candidate gene identification and diagnostic marker verification

The KASP markers associated with the candidate region were utilized to genotype the 218 pea individuals with known phenotypes (Table S1). Markers associated with genotypes in susceptible individuals that correspond to those of the resistant parent would be removed. The applicable markers were utilized as diagnostic markers for the detection of the gene in pea accessions. The candidate resistance gene could be refined to those linked with these diagnostic markers. The diagnostic markers developed were also validated using individual plants from the CW9 pea lines.

### Candidate gene cloning and sequence alignment

The candidate gene *Psat4g213640* identified through haplotype analysis were further analyzed by sequence alignment. The primers *PsFwC9*-gene-F/R were designed with the NCBI primer designing tool (https://www.ncbi.nlm.nih.gov/tools/primer-blast/) to amplify *Psat4g213640* from the parental lines CW9-1 and CW9-8 (Table S2). The PCR amplification was completed through a TC-E-480 thermocycler (Bioer Technology, Hangzhou) in 40-μl reaction mixtures: containing 4 μl of genomic DNA (~20 ng/μl), 1.6 μl of each primer, 20 μl of 2× Taq PCR Master Mix (Tiangen Biotech) and 12.8 μl of ddH_2_O. The thermal cycling conditions were 94°C for 5 min; followed by 40 cycles of 94°C for 30 s, 55°C for 30 s and 72°C for 1 min, with a final extension at 72°C for 10 min. The PCR products were purified using the TIANquick Midi Purification Kit (TIANGEN, Beijing, China) and sequenced by Sangon Biotech (Shanghai, China). The resulting nucleotide sequences and their predicted amino acid sequences were aligned and analyzed against the corresponding genes in the reference genome Caméor using DNAMAN v9.0.1.116 (Lynnon Biosoft, PQ, Canada). Moreover, the protein structure of the candidate gene was predicted using the online tool AlphaFold 3 (https://alphafoldserver.com/) and visualized with ChimeraX v1.9 (https://www.cgl.ucsf.edu/chimerax/).

### Quantitative real-time PCR

The total RNA was extracted from the roots of parental plants at 0, 12, 24, 48 and 96 h after inoculation, as well as from the root, stem and leaf tissues of uninoculated parental plants in three biological replicates. Following the manufacturer′s protocol, total RNA was extracted with the FastPure Universal Plant Total RNA Isolation Kit (Vazyme Biotech, Nanjing, China), and first-strand cDNA was synthesized using the RT-PCR system for EasyScript First-Strand cDNA Synthesis SuperMix kit (TransGen Biotech, Beijing, China). The qRT-PCR primer pair *PsFwC9*-RT-4F/R for *Psat4g213640* was designed by the NCBI primer designing tool, and the *PsActin* was employed as an internal control for transcriptional normalization following the recommendations of Die *et al.* [48] (Table S3). The qRT-PCR reactions were conducted on a QuantStudio™ 5 Real-Time PCR Instrument (Thermo Fisher Scientific, Waltham, MA, USA) with the reaction mixtures: 2 μl of cDNA (~100 ng/μl), 0.4 μl of each primer, 10 μl of 2× *TransStart*® Top Green qPCR SuperMix and 7.2 μl of ddH_2_O. The qRT-PCR reactions were performed on an ABI 7500 Real-Time PCR System (Applied Biosystems, USA) using the following program: 50°C for 2 min, 95°C for 10 min, followed by 40 cycles of 95°C for 5 s and 60°C for 34 s. The relative expression levels were calculated and analyzed using the 2^−ΔΔCt^ method [49].

### *Agrobacterium rhizogenes*-mediated transformation of seedlings and inoculation identification

The full-length cDNA of *PsFwC9* candidate gene *Psat4g213640* was amplified with primer pair OE-*PsFwC9*-F/R (Table S3), incorporating NcoI and BstEII restriction sites, and then inserted into the pCAMBIA3301 vector to create the overexpression (OE) vector *PsFwC9*-OE [50]. For RNAi vector, a fragment was sequentially amplified and modified with XbaI/SmaI and SpeI/SacI restriction enzymes, comprising the sense and antisense sequences of *PsFwC9*, and then ligated into the pROKII vector to generate the recombinant RNAi vector *PsFwC9*-RNAi [50]. The pCAMBIA3301 and pROKII vectors were used as controls (CK). The parental lines CW9-1 (for OE) and CW9-8 (for RNAi) were used for transformation with *A. rhizogenes* strain K599. Transgenic pea hairy root composite plants were generated using a slightly modified method based on that described by Fan *et al.* [57] for soybean. After hairy roots developed for 14 days, these seedlings with hairy roots were thoroughly washed, with one-third of the hairy roots removed, and then immersed in a microconidia suspension of 1.0 × 10^7^ microconidia/ml. After 24 h of inoculation, the longest and strongest hairy root, ~2 cm in length, was collected from the seedlings. About 1 cm of the collected root was sent to Solarbio (Beijing, China) for PAS staining, and the visualization was performed using SlideViewer v2.7 software (3DHistech, Budapest, Hungary), while the remainder was used to assess the expression levels of *Psat4g213640*, following the same method as qRT-PCR method described above. The seedlings were then transplanted into new cups for continued observation of their phenotypes. All experiments, including overexpression, gene silencing and subsequent inoculation, were conducted in three independent biological replicates.

### Subcellular localization

The full-length cDNA of *Psat4g213640* in CW9-8, containing HindIII and SpeI restriction sites, was amplified using the SL-PsFwC9-F/R primer pair (Table S3). The amplified fragment was inserted into the modified pCAMBIA1300-GFP expression vector [50] to generate the 35S::PsFwC9^CW9-8^-GFP and 35S::PsFwC9^CW9-1^-GFP fusion constructs. The recombinant plasmids were introduced into *A. tumefaciens* strain GV3101 and used for transient expression in *N. benthamiana* leaves, with pCAMBIA1300-GFP serving as the control [82]. The tobacco seedlings were kept in the dark for 24 h prior to infiltration, and the leaves were infiltrated with *Agrobacterium* suspensions and incubated for 48 h under suitable growth conditions. Fluorescence signals were observed using an LSM900 confocal laser scanning microscope (Zeiss, Oberkochen, Germany). Based on the preliminary localization pattern observed in the transient expression assay, co-localization analysis was further performed using the corresponding subcellular marker to verify the subcellular localization of *PsFwC9*.

## Supplementary Material

Web_Material_uhag166

## Data Availability

The raw sequence data reported in this paper have been deposited in the Genome Sequence Archive (Genomics, Proteomics & Bioinformatics 2025) in National Genomics Data Center (Nucleic Acids Res 2025), China National Center for Bioinformation/Beijing Institute of Genomics, Chinese Academy of Sciences (GSA: CRA033056), which are publicly accessible at https://ngdc.cncb.ac.cn/gsa.

## References

[1] Cousin R . Peas (*Pisum sativum* L.). Field Crop Res. 1997;53:111–30

[2] Dahl WJ, Foster LM, Tyler RT. Review of the health benefits of peas (*Pisum sativum L.*). Br J Nutr. 2012;108:S3–10 10.1017/S0007114512000852 22916813

[3] Guindon MF, Cazzola F, Palacios T. et al. Biofortification of pea (*Pisum sativum L.*): a review. J Sci Food Agric. 2021;101:3551–63 33417241 10.1002/jsfa.11059

[4] Wu DT, Li WX, Wan JJ. et al. A comprehensive review of pea (*Pisum sativum L.*): chemical composition, processing, health benefits, and food applications. Foods. 2023;12:2527 37444265 10.3390/foods12132527 PMC10341148

[5] Li L, Yang T, Liu R. et al. Food legume production in China. Crop J. 2017;5:115–26

[6] Woo SL, De Filippis F, Zotti M. et al. Pea-wheat rotation affects soil microbiota diversity, community structure, and soilborne pathogens. Microorganisms. 2022;10:370 35208825 10.3390/microorganisms10020370 PMC8876268

[7] Tulbek MC, Wang YL, Hounjet M. Pea—A sustainable vegetable protein crop. In: Sudarshan N, Wanasundara JPD, Scanlin L. et al., eds. Sustainable Protein Sources. Elsevier: New York, 2024,143–62

[8] El-Sharkawy HHA, Abbas MS, Soliman AS. et al. Synergistic effect of growth-promoting microorganisms on bio-control of *Fusarium oxysporum* f. sp. *pisi*, growth, yield, physiological and anatomical characteristics of pea plants. Pestic Biochem Physiol. 2021;178:104939 34446206 10.1016/j.pestbp.2021.104939

[9] Sharma A, Rathour R, Plaha P. et al. Induction of Fusarium wilt (*Fusarium oxysporum* f. sp. *pisi*) resistance in garden pea using induced mutagenesis and in vitro selection techniques. Euphytica. 2010;173:345–56

[10] Deng D, Sun S, Wu W. et al. Screening for pea germplasms resistant to Fusarium wilt race 5. Agronomy. 2022;12:1354

[11] Kraft JM, Pfleger FL. Compendium of Pea Diseases and Pests. 2nd ed. St. Paul, MN, USA: American Phytopathological Society; 2001:13–4

[12] Jha UC, Bohra A, Pandey S. et al. Breeding, genetics, and genomics approaches for improving Fusarium wilt resistance in major grain legumes. Front Genet. 2020;11:1001 33193586 10.3389/fgene.2020.01001 PMC7644945

[13] Sampaio AM, Araujo SD, Rubiales D. et al. Fusarium wilt management in legume crops. Agronomy. 2020;10:1073

[14] Neumann S, Xue AG. Reactions of field pea cultivars to four races of *Fusarium oxysporum* f. sp. *pisi*. Can J Plant Sci. 2003;83:377–9

[15] Bani M, Rubiales D, Rispail N. et al. A detailed evaluation method to identify sources of quantitative resistance to *Fusarium oxysporum* f. sp. *pisi* race 2 within a *Pisum* spp. germplasm collection. Plant Pathol. 2012;61:532–42

[16] Deng D, Sun S, Wu W. et al. Fine mapping and identification of a Fusarium wilt resistance gene *FwS1* in pea. Theor Appl Genet. 2024;137:171 38918246 10.1007/s00122-024-04682-1

[17] Jain S, Weeden NF, Kumar A. et al. Functional codominant marker for selecting the *Fw* gene conferring resistance to Fusarium wilt race 1 in pea. Crop Sci. 2015;55:2639–46

[18] McPhee KE, Inglis DA, Gundersen B. et al. Mapping QTL for Fusarium wilt race 2 partial resistance in pea (*Pisum sativum*). Plant Breed. 2012;131:300–6

[19] Kwon SJ, Smýkal P, Hu J. et al. User-friendly markers linked to Fusarium wilt race 1 resistance *Fw* gene for marker-assisted selection in pea. Plant Breed. 2013;132:642–8

[20] Coyne CJ, Inglis DA, Whitehead SJ. et al. Chromosomal location of *Fwf*, the Fusarium wilt race 5 resistance gene in *Pisum sativum*. Pisum Genet. 2000;32:20–2

[21] Haglund WA, Kraft JM. *Fusarium oxysporum* f. sp. *pisi*, race 6: occurrence and distribution. Phytopathology. 1979;81:818–20

[22] Okubara PA, Inglis DA, Muehlbauer FJ. et al. A novel RAPD marker linked to the Fusarium wilt race 5 resistance gene (*Fwf*) in *Pisum sativum*. Pisum Genet. 2002;34:6–8

[23] Rozhmina TA, Kanapin AA, Bankin MP. et al. Identification of two QTLs controlling flax resistance to Fusarium wilt. Biophysics. 2024;69:57–62

[24] Tassone MR, Bagnaresi P, Desiderio F. et al. A genomic BSA-seq approach for the characterization of QTLs underlying resistance to *Fusarium oxysporum* in eggplant. Cells. 2022;11:2548 36010625 10.3390/cells11162548 PMC9406753

[25] Van Ooijen G, Mayr G, Kasiem MM. et al. Structure-function analysis of the NB-ARC domain of plant disease resistance proteins. J Exp Bot. 2008;59:1383–97 18390848 10.1093/jxb/ern045

[26] Shokouhifar F, Mamarabadi M, Khyrabad MM. et al. Tracking of the gene Fom2 and study on the genetic diversity of NB-ARC domain in the number of resistant and sensitive melon cultivars against *Fusarium oxysporum* f. sp. *melonis* (race 1). Australas Plant Pathol. 2016;45:279–88

[27] Wu X, Li N, Hao J. et al. Genetic diversity of Chinese and global pea (*Pisum sativum L.*) collections. Crop Sci. 2017;57:1574–84

[28] Chen HL, Tian J, Zhu ZD. et al. Current status and future prospective of food legumes production and seed industry in China. Sci Agric Sin. 2021;54:493–503 (In Chinese)

[29] FAO (Food and Agriculture Organization) . Online statistical database: Crops and livestock products. 2023 (Accessed June 11, 2025). https://www.fao.org/faostat/en/#data/QCL

[30] Zhang J . Rates of conservative and radical nonsynonymous nucleotide substitutions in mammalian nuclear genes. J Mol Evol. 2000;50:56–68 10654260 10.1007/s002399910007

[31] Wang M, Wang D, Yu J. et al. Enrichment in conservative amino acid changes among fixed and standing missense variations in slowly evolving proteins. PeerJ. 2020;8:e9983 32995099 10.7717/peerj.9983 PMC7501800

[32] Michielse CB, Rep M. Pathogen profile update: *Fusarium oxysporum*. Mol Plant Pathol. 2009;10:311–24 19400835 10.1111/j.1364-3703.2009.00538.x PMC6640313

[33] Ma LJ, Van Der Does HC, Borkovich KA. et al. Comparative genomics reveals mobile pathogenicity chromosomes in *Fusarium*. Nature. 2010;464:367–73 20237561 10.1038/nature08850 PMC3048781

[34] Taylor A, Vágány V, Jackson AC. et al. Identification of pathogenicity-related genes in *Fusarium oxysporum* f. sp. *cepae*. *Mol*. Plant Pathol. 2016;17:1032–47 10.1111/mpp.12346 PMC4982077 26609905

[35] Dita M, Barquero M, Heck D. et al. Fusarium wilt of banana: current knowledge on epidemiology and research needs toward sustainable disease management. Front Plant Sci. 2018;9:1468 30405651 10.3389/fpls.2018.01468 PMC6202804

[36] Joshi RK, Nayak S. Gene pyramiding—a broad spectrum technique for developing durable stress resistance in crops. Biotechnol Mol Biol Rev. 2010;5:51–60

[37] Dormatey R, Sun C, Ali K. et al. Gene pyramiding for sustainable crop improvement against biotic and abiotic stresses. Agronomy. 2020;10:1255

[38] Michelmore RW, Paran I, Kesseli RV. Identification of markers linked to disease-resistance genes by bulked segregant analysis: a rapid method to detect markers in specific genomic regions by using segregating populations. Proc Natl Acad Sci U S A. 1991;88:9828–32 1682921 10.1073/pnas.88.21.9828 PMC52814

[39] Takagi H, Abe A, Yoshida K. et al. QTL-seq: rapid mapping of quantitative trait loci in rice by whole genome resequencing of DNA from two bulked populations. Plant J. 2013;74:174–83 23289725 10.1111/tpj.12105

[40] Sugihara Y, Young L, Yaegashi H. et al. High-performance pipeline for MutMap and QTL-seq. PeerJ. 2022;10:e13170 35321412 10.7717/peerj.13170 PMC8935991

[41] Rubiales D, González-Bernal MJ, Warkentin T. et al. Advances in pea breeding. In: Hochmuth G, ed. Achieving Sustainable Cultivation of Vegetables. Burleigh Dodds Science Publishing: Cambridge, UK, 2019,575–606

[42] Zhang B, Qi F, Hu G. et al. BSA-seq-based identification of a major additive plant height QTL with an effect equivalent to that of *Semi-dwarf 1* in a large rice F_2_ population. Crop J. 2021;9:1428–37

[43] Wang Y, Teng Z, Li H. et al. An activated form of NB-ARC protein *RLS1* functions with cysteine-rich receptor-like protein *RMC* to trigger cell death in rice. Plant Commun. 2023;4:100459 36203361 10.1016/j.xplc.2022.100459 PMC10030324

[44] Beniwal D, Dhall RK, Vikal Y. et al. BSA-seq identifies a major locus on chromosome-6 for rust (*Uromyces viciae-fabae*) resistance in garden pea (*Pisum sativum L.*). Nucleus. 2024;1:1–11

[45] Beji S, Fontaine V, Devaux R. et al. Genome-wide association study identifies favorable SNP alleles and candidate genes for frost tolerance in pea. BMC Genomics. 2020;21:1–21 10.1186/s12864-020-06928-w PMC7430820 32753054

[46] Zheng Y, Xu F, Li Q. et al. QTL mapping combined with bulked segregant analysis identify SNP markers linked to leaf shape traits in *Pisum sativum* using SLAF sequencing. Front Genet. 2018;9:615 30568674 10.3389/fgene.2018.00615 PMC6290080

[47] Zhang P, Wang Y, Sun T. et al. Fine mapping *PsPS1*, a gene controlling pod softness that defines market type in pea (*Pisum sativum*). Plant Breed. 2022;141:418–28

[48] Die JV, Román B, Nadal S. et al. Evaluation of candidate reference genes for expression studies in *Pisum sativum* under different experimental conditions. Planta. 2010;232:145–53 20379832 10.1007/s00425-010-1158-1

[49] Livak KJ, Schmittgen TD. Analysis of relative gene expression data using real-time quantitative PCR and the 2^−ΔΔCt^ method. Methods. 2001;25:402–8 11846609 10.1006/meth.2001.1262

[50] Wu L, Chang Y, Wang L. et al. The aquaporin gene *PvXIP1;2* conferring drought resistance identified by GWAS at seedling stage in common bean. Theor Appl Genet. 2022;135:485–500 34698878 10.1007/s00122-021-03978-w

[51] Schmittgen TD, Livak KJ. Analyzing real-time PCR data by the comparative CT method. Nat Protoc. 2008;3:1101–8 18546601 10.1038/nprot.2008.73

[52] Ye J, Jin CF, Li N. et al. Selection of suitable reference genes for qRT-PCR normalisation under different experimental conditions in *Eucommia ulmoides* Oliv. Sci Rep. 2018;8:15043 30301911 10.1038/s41598-018-33342-w PMC6177395

[53] Bartholomew ES, Xu S, Zhang Y. et al. A chitinase *CsChi23* promoter polymorphism underlies cucumber resistance against *Fusarium oxysporum* f. sp. *cucumerinum*. New Phytol. 2022;236:1471–86 36068958 10.1111/nph.18463

[54] Dong C, Zhang L, Zhang Q. et al. *Tiller Number1* encodes an ankyrin repeat protein that controls tillering in bread wheat. Nat Commun. 2023;14:836 36788238 10.1038/s41467-023-36271-z PMC9929037

[55] Huang S, Wang J, Song R. et al. Balanced plant helper NLR activation by a modified host protein complex. Nature. 2025;639:447–55 39939760 10.1038/s41586-024-08521-7

[56] Miao P, Wang H, Wang W. et al. A widespread plant defense compound disarms bacterial type III injectisome assembly. Science. 2025;387:eads0377 40014714 10.1126/science.ads0377

[57] Fan YL, Zhang XH, Zhong LJ. et al. One-step generation of composite soybean plants with transgenic roots by *Agrobacterium rhizogenes*-mediated transformation. BMC Plant Biol. 2020;20:208 32397958 10.1186/s12870-020-02421-4 PMC7333419

[58] Kereszt A, Li D, Indrasumunar A. et al. *Agrobacterium rhizogenes*-mediated transformation of soybean to study root biology. Nat Protoc. 2007;2:948–52 17446894 10.1038/nprot.2007.141

[59] Georgiev M, Agostini E, Ludwig-Müller J. et al. Genetically transformed roots: from plant disease to biotechnological resource. Trends Biotechnol. 2012;30:528–37 22906523 10.1016/j.tibtech.2012.07.001

[60] Yu WC, Fersht AR. Stability and solvation of Thr/Ser to ala and Gly mutations at the N-cap of α-helices. FEBS Lett. 1994;347:304–9 8034023 10.1016/0014-5793(94)00574-5

[61] Wang W, Hu B, Li A. et al. *NRT1.1s* in plants: functions beyond nitrate transport. J Exp Bot. 2020;71:4373–9 31832669 10.1093/jxb/erz554 PMC7382373

[62] Sun J, Bankston JR, Payandeh J. et al. Crystal structure of the plant dual-affinity nitrate transporter *NRT1.1*. Nature. 2014;507:73–7 24572362 10.1038/nature13074 PMC3968801

[63] Liu KH, Liu M, Lin Z. et al. NIN-like protein 7 transcription factor is a plant nitrate sensor. Science. 2022;377:1419–25 36137053 10.1126/science.add1104 PMC9628810

[64] Nguyen TA, Lee C. Thr-to-Ala mutation leads to a larger aromatic pair and reduced packing density in α1, α3-helices during thioredoxin cold adaptation. ACS Omega. 2024;9:10812–24 38463323 10.1021/acsomega.3c09806 PMC10918799

[65] Eichmann R, Schäfer P. The endoplasmic reticulum in plant immunity and cell death. Front Plant Sci. 2012;3:200 22936941 10.3389/fpls.2012.00200 PMC3424470

[66] Saijo Y . ER quality control of immune receptors and regulators in plants. Cell Microbiol. 2010;12:716–24 20408850 10.1111/j.1462-5822.2010.01472.x

[67] Bi W, Chen Y, Song Y. et al. Potato DMP2 positively regulates plant immunity by modulating endoplasmic reticulum homeostasis. J Integr Plant Biol. 2025;67:1568–81 40052459 10.1111/jipb.13876

[68] Groenendyk J, Agellon LB, Michalak M. Calcium signaling and endoplasmic reticulum stress. Int Rev Cell Mol Biol. 2021;363:1–20 34392927 10.1016/bs.ircmb.2021.03.003

[69] Krebs J, Agellon LB, Michalak M. Ca^2+^ homeostasis and endoplasmic reticulum (ER) stress: an integrated view of calcium signaling. Biochem Biophys Res Commun. 2015;460:114–21 25998740 10.1016/j.bbrc.2015.02.004

[70] Cao J, Wang C, Hao N. et al. Endoplasmic reticulum stress and reactive oxygen species in plants. Antioxidants. 2022;11:1240 35883731 10.3390/antiox11071240 PMC9311536

[71] Eisner V, Csordás G, Hajnóczky G. Interactions between sarco-endoplasmic reticulum and mitochondria in cardiac and skeletal muscle–pivotal roles in Ca^2+^ and reactive oxygen species signaling. J Cell Sci. 2013;126:2965–78 23843617 10.1242/jcs.093609 PMC3711195

[72] Chen S, Zhou Y, Chen Y. et al. Fastp: an ultra-fast all-in-one FASTQ preprocessor. Bioinformatics. 2018;34:i884–90 30423086 10.1093/bioinformatics/bty560 PMC6129281

[73] Vasimuddin M, Misra S, Li H. et al. Efficient architecture-aware acceleration of BWA-MEM for multicore systems. In: 2019 IEEE Parallel and Distributed Processing Symposium (IPDPS). IEEE, 2019;314–24

[74] Kreplak J, Madoui MA, Cápal P. et al. A reference genome for pea provides insight into legume genome evolution. Nat Genet. 2019;51:1411–22 31477930 10.1038/s41588-019-0480-1

[75] Danecek P, Bonfield JK, Liddle J. et al. Twelve years of SAMtools and BCFtools. Gigascience. 2021;10:giab008 33590861 10.1093/gigascience/giab008 PMC7931819

[76] Van der Auwera GA, O’Connor BD. Genomics in the Cloud: Using Docker, GATK, and WDL in Terra. Sebastopol, CA, USA: O’Reilly Media; 2020:

[77] Poplin R, Ruano-Rubio V, DePristo MA. et al. Scaling accurate genetic variant discovery to tens of thousands of samples. *bioRxiv*. 2018;201178

[78] Danecek P, Auton A, Abecasis G. et al. The variant call format and VCFtools. Bioinformatics. 2011;27:2156–8 21653522 10.1093/bioinformatics/btr330 PMC3137218

[79] Cingolani P, Platts A, Wang LL. et al. A program for annotating and predicting the effects of single nucleotide polymorphisms, SnpEff: SNPs in the genome of *Drosophila melanogaster* strain w1118; iso-2; iso-3. Fly. 2012;6:80–92 22728672 10.4161/fly.19695 PMC3679285

[80] Chen C, Wu Y, Li J. et al. TBtools-II: a “one for all, all for one” bioinformatics platform for biological big-data mining. Mol Plant. 2023;16:1733–42 37740491 10.1016/j.molp.2023.09.010

[81] Wood DE, Lu J, Langmead B. Improved metagenomic analysis with kraken 2. Genome Biol. 2019;20:257 31779668 10.1186/s13059-019-1891-0 PMC6883579

[82] Sheludko YV, Sindarovska YR, Gerasymenko IM. et al. Comparison of several *Nicotiana* species as hosts for high-scale *Agrobacterium*-mediated transient expression. Biotechnol Bioeng. 2007;96:608–14 16983697 10.1002/bit.21075

