# Supplementary material for: Identification and characterization of PsFwC9 conferring Fusarium wilt resistance in pea
Source: Hortic Res. 2026 Apr 23;13(9):uhag166. doi: 10.1093/hr/uhag166 (PMC13532407; doi:10.1093/hr/uhag166)
Supplement: Supplementary file 1 [file web_material_uhag166.zip › Fig S1.pdf]

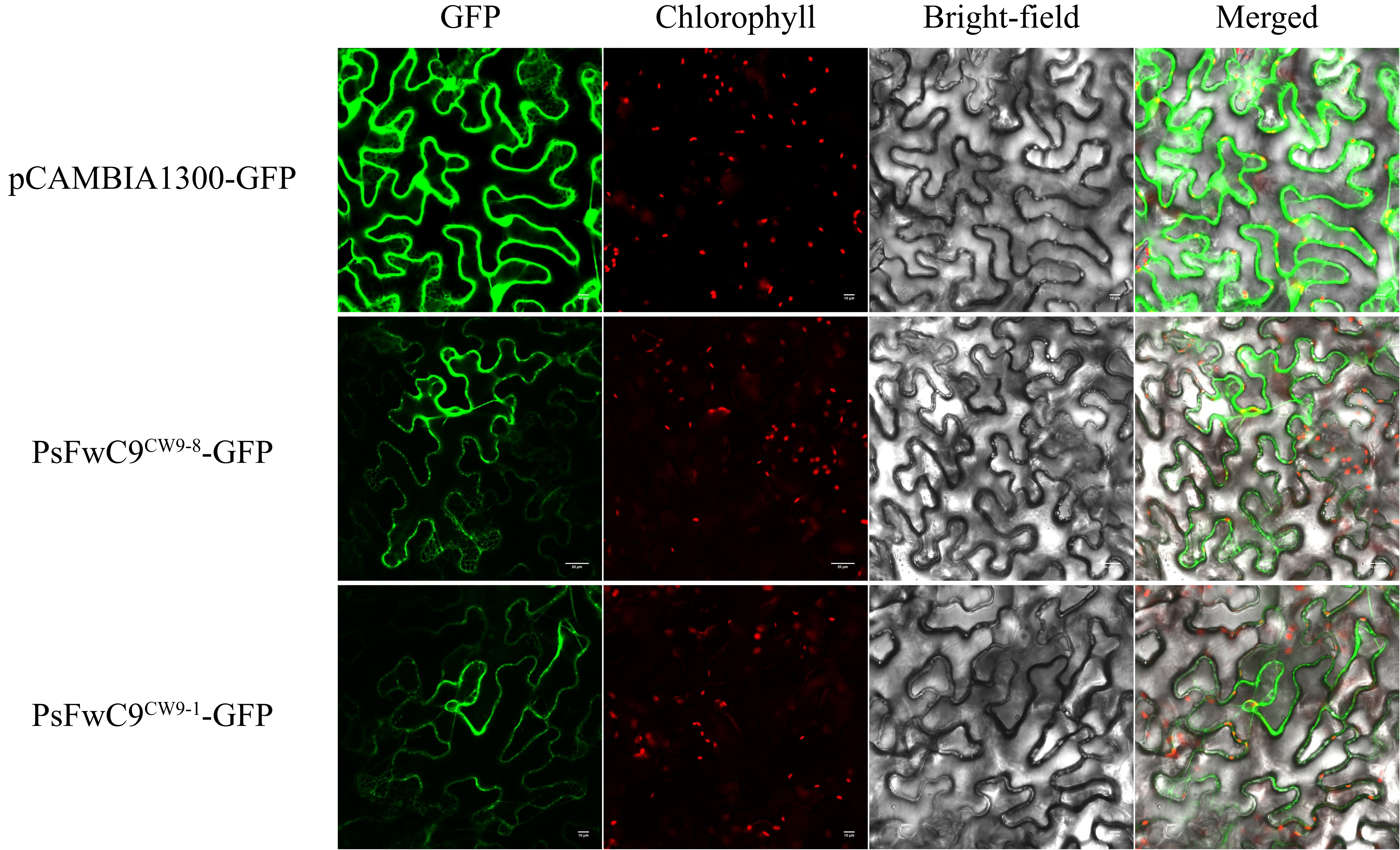
GFP
Chlorophyll
Bright-field
Merged
pCAMBIA1300-GFP
PsFwC9CW9-8-GFP
PsFwC9CW9-1-GFP
